# Urolithin A Promotes Angiogenesis and Tissue Regeneration in a Full-Thickness Cutaneous Wound Model

**DOI:** 10.3389/fphar.2022.806284

**Published:** 2022-03-14

**Authors:** Zhen-hua Feng, Jia Chen, Pu-tao Yuan, Zhong-yin Ji, Si-yue Tao, Lin Zheng, Xiao-an Wei, Ze-yu Zheng, Bing-jie Zheng, Bin Chen, Jian Chen, Feng-dong Zhao

**Affiliations:** ^1^ Department of Orthopaedic Surgery, Sir Run Run Shaw Hospital, Zhejiang University School of Medicine, Hangzhou, China; ^2^ Key Laboratory of Musculoskeletal System Degeneration and Regeneration Translational Research of Zhejiang Province, Hangzhou, China; ^3^ Department of Orthopedics, The Second Affiliated Hospital of Jiaxing University, Jiaxing, China

**Keywords:** urolithin A, wound healing, PI3K/AKT pathway, angiogenesis, docking

## Abstract

The treatment of chronic wound is an important topic of current clinical issue. Neovascularization plays a crucial role in skin wound healing by delivering fresh nutrients and oxygen to the wound area. The aim of this study was to investigate the mechanisms of urolithin A (UA) in angiogenesis during wound healing. The results of *in vitro* experiments showed that treatment with UA (5–20 μM) promoted the proliferation, migration, and angiogenic capacity of HUVECs. Furthermore, we investigated the effect of UA *in vivo* using a full-thickness skin wound model. Subsequently, we found that UA promoted the regeneration of new blood vessels, which is consistent with the results of accelerated angiogenesis *in vitro* experiments. After UA treatment, the blood vessels in the wound are rapidly formed, and the deposition and remodeling process of the collagen matrix is also accelerated, which ultimately promotes the effective wound healing. Mechanistic studies have shown that UA promotes angiogenesis by inhibiting the PI3K/AKT pathway. Our study provides evidence that UA can promote angiogenesis and skin regeneration in chronic wounds, especially ischemic wounds.

## Introduction

Skin wounds remain a major global public health problem due to the increasing number of burns, trauma, and chronic diseases and can result in pain, infection, and even amputation (Valencia et al., 2001; Eberhardt and Raffetto, 2014). Wound healing is a complex process involving a variety of growth factors, cells, and extracellular matrix (ECM), usually divided into three stages: inflammation, proliferation, and remodeling (Hosemann et al., 1991; Watelet et al., 2002). Surgical debridement and auxiliary wound care methods are the conventional treatment of skin wounds (Bennett et al., 2003). However, the new vessel and granulation tissue are easily destroyed, causing bleeding and secondary injury, which not only is unfavorable for wound healing but also results in unbearable pain. It is necessary to find a less invasive and effective method.

Angiogenesis is a key factor in wound healing caused by chronic and ischemic injuries (Folkman, 1995). New blood vessels are essential for wound healing, providing nutrients and oxygen to cells at the wound site. Endothelial cells are known to be key cells in angiogenesis, which are responsible for many biological activities such as proliferation, adhesion, and transport from pre-existing blood vessels. Various growth factors have been shown to promote endothelial cell proliferation, migration, differentiation, and angiogenesis, such as the vascular endothelial cell growth factor (VEGF) and basic fibroblast growth factor (bFGF), which have been used in the treatment of ischemic diseases (Scharpfenecker et al., 2007; Bir et al., 2014). However, the high price, rapid degradation, anaphylaxis, and other adverse side effects of these growth factors limit their clinical application potential. Therefore, finding an affordable, less complication-promoting drug that promotes angiogenesis in skin wounds may be a better alternative solution.

Urolithin A (UA) is one of the metabolites of ellagitannins and ellagic acid extracted from pomegranates and other fruits and nuts, which have a wide range of functions including anti-inflammatory (Y. Zhang et al., 2021) and anticancer effects (Cheng et al., 2021; El-Wetidy et al., 2021). The previous study reported that UA augments angiogenesis in C2C12 cells (Ghosh et al., 2020). PI3K, AKT, mTOR, and other signaling proteins participate in the wound-healing process (X. Dong et al., 2020; Wang et al., 2018a). Interestingly, UA inhibited the PI3K/AKT-dependent signaling pathway in chondrocytes and mice osteoarthritis model (Fu et al., 2019). However, the potential vascular regulation and wound-healing effects of UA are unclear.

In the present study, we hypothesized that UA could activate the angiogenic capacity of HUVECs and accelerate wound healing. The mechanism of pro-angiogenic UA in HUVECs was investigated. A full-thickness skin wound model was used to study the therapeutic efficacy of UA.

## Materials and Methods

### Ethical Approval

All experiments were carried out under the guidelines of the Ethics Committee of Sir Run Run Shaw Hospital and in compliance with the Guidelines for Care and Use of Laboratory Animals published by the National Institutes of Health.

### Reagents

Urolithin A (purity ≥98%) was purchased from MCE (Shanghai, China). Crystal violet and DAPI were purchased from Beyotime (Shanghai, China). DMSO was purchased from Sigma-Aldrich (St. Louis, MO, United States). Cell Counting Kit-8 (CCK-8) was purchased from MCE (Shanghai, China).

### Cell Culture

HUVECs were obtained from iCell Bioscience (Shanghai, China). We maintained cells at 37°C in RMPI 1640 containing 10% fetal bovine serum. HUVECs were cultured at 37°C in a humidifying incubator under 5% CO2 atmosphere. The first 20 passages of cells were used for the experiment.

### Transwell Migration Test

The Transwell chamber (BD Biosciences) was used for Transwell migration. For the migration assay, a one-day incubation process was performed in 100 μL serum-free medium. 500 μL of complete medium (containing FBS and 1640 medium) was placed in the lower chamber of the Transwell chamber and 5 × 10^4^ cells in the upper chamber. An inverted light microscope (Zeiss, Primovert) was used to generate representative images.

### CCK-8 Assay

The cell viability exhibited by HUVECs with different doses of UA (0–40 μM) was detected by the CCK-8 assay. Briefly, HUVECs were incubated with 5 × 10^3^ cells in the 96-well plates for 48 h. The cells were added with 100 μL RMPI 1640 including 10% tetrazolium substrate and incubated for 1 h. Then, we use a spectrophotometer to measure the absorbance value at 450 nm.

### *In Vitro* Tube Formation Assay

A tube-forming experiment was performed on Matrigel glue to evaluate the effect of UA on the morphogenesis and tube-forming ability of HUVECs. Simply put, the matrix gel solution was melted overnight at 4°C, then placed in a µ-slide, and placed in a cell incubator for 1 h to solidify. 5,000 HUVECs pretreated with different concentrations of UA (0, 5, 10, and 20 µM) were seeded on the pre-coated µ-slide of the matrix. Observing the formation of the tube, the average of five independent fields was counted under a Nikon inverted optical microscope.

### Western Blotting Analysis

Firstly, HUVECs were plated in a six-well plate and cultured at 40–60% density. After HUVECs were fully attached, they were exposed to different treatments for 48 h. Next, we use radioimmunoprecipitation analysis (RIPA) lysis buffer which contains protease inhibitors and phosphatase inhibitors to lyse HUVECs for 30 min. The lysate is collected and centrifuged for 15 min at 14000 g at 4°C. The supernatant is collected and mixed with 5× loading buffer. It is put in a metal bath at 100°C for 10 min. Next, we perform WB experiments. SDS-PAGE (10%) gel is used to separate the proteins and transfer them to 0.45-μm PVDF membranes. 5% skimmed milk powder is used to seal the PVDF membrane for 1 h at room temperature. The membrane is cut according to the corresponding molecular weight, and then the specific primary antibody is used: anti-AKT (1:1,000), anti-p-AKT (1:1,000), anti-PI3K (1:1,000), and anti-p-PI3K (1:1,000), to incubate overnight at 4°C. The next day, the PVDF membrane was washed three times with TBST buffer and then incubated with the corresponding secondary antibody for 1 h at room temperature. Then, the PVDF membrane was washed three times again with TBST buffer. We use the GE imaging system to observe and store protein bands.

### Immunofluorescence Staining

Immunofluorescence was used to evaluate protein expression levels. The cells were fixed with paraformaldehyde and permeabilized with 0.3% Triton X-100 and incubated with the CD31 antibody (1:200) at 4°C. Then, a fluorescein-conjugated secondary antibody was added to the cells, which were incubated for 1 h at 4°C in the dark, followed by a counterstaining process of 15 min with DAPI at room temperature in the dark to stain the nuclei. A Nikon A1 microscope (Nikon, Japan) with a digital camera was used to capture fluorescence images.

### EdU Assay

EdU assay was used EdU kit (Beyotime) according to the protocol. Firstly, we seeded HUVECs in 12-well plates. After adherence, HUVECs were starved for 8 h with FBS-free 1640 to synchronize the cell cycle. Afterward, the cells were treated with different doses of UA for 24 h. The cell culture medium was then replaced with fresh 1640 containing EdU and incubated for 2 h. Next, we fix the cells with 4% paraformaldehyde for 15 min and then treat them with 0.3% Triton X-100 for 20 min. After that, the medium of each well was replaced with fresh 1640 containing 10 μM EdU and cultured at 37°C for 2 h. Then, 4% paraformaldehyde was used to fix the cells for 15 min, and the fixed cells received 20 min of incubation with 0.3% Triton X-100. Afterward, they were incubated for 25 min in Click reaction buffer in the dark. The nucleus is stained with DAPI. A Nikon A1 microscope (Nikon, Japan) with a digital camera was used to capture fluorescence images.

### Molecular Docking

We choose AKT (PDB ID: 3QKK) and PI3K (PDB ID: 5ITD) for molecular docking research (Kallan et al., 2011; Hoegenauer et al., 2016). Both protein structures are downloaded from the PDB (https://www.rcsb.org/) and docked. AutoDockTools (version 1.5.6) was used for protein–ligand docking analysis. This tool can provide the flexibility of binding the ligand to the residues of the binding pocket. The final image is generated by UCSF PyMoL.

### Establishment of a Wound Model

Twenty-four Sprague Dawley (SD) rats were anesthetized with pentobarbital and placed in the prone position. After the rat’s back hair was shaved and disinfected, two full-thickness wounds with a diameter of approximately 20 mm were made on both sides by using a 20 mm diameter mold and scissors. The UA group was intragastrically administered at a dose of 25 mg/kg/d until 21 days after surgery (Lin et al., 2020). All rats were placed in separate cages to prevent each other from biting the wounds. Pictures of the wounds were taken roughly every 7 days. The wound area was evaluated as a percentage of the original wound area by using Image-Pro Plus 6.0 software. At different endpoint times, rats were euthanized by intraperitoneal injection of an overdose of pentobarbital.

### Histology Analysis and Immunohistochemistry

To prepare the animal tissue samples, we embedded the formalin-fixed tissue samples in paraffin, cut them into 5 μm sections, and placed them on slides. For immunohistochemical staining, the slides were dewaxed in xylene and rehydrated with graded alcohol. Then, the slides were incubated in 3% hydrogen peroxide to impede endogenous peroxidase activity. The slides were boiled for 30 min in 10 mM sodium citrate (pH 6.0) for antigen retrieval, blocked with 5% normal goat serum for 15 min, and then incubated with the indicated antibodies: collagen I (1:300) or anti-collagen III (1:300), overnight at 4°C in a humid room. The next day, the slides were incubated with the secondary antibody for 1 h at room temperature after the PBS washing process. A Metal Enhanced DAB Substrate Kit (Solarbio Life Sciences, Peking, China) was used to detect immunoreactivity.

### Masson’s Trichrome and Hematoxylin and Eosin Staining

Masson’s trichrome staining: The slices were dewaxed. Next, we stained slices with the Weigert iron hematoxylin staining solution for 5 min. Subsequently, we stained slices with Masson blue liquid for 5 min and with Ponceau red fuchsin staining for 5 min and aniline blue staining for 2 min. Finally, the slices were dehydrated and mounted. Hematoxylin and eosin staining: Firstly, the slices were dewaxed and stained with hematoxylin for 2 min. The slices were soaked in the differentiation solution for 3 min. Next, the slices were dyed with eosin dye solution for 1 min.

### Statistical Analysis

Prism 8 (GraphPad Software, United States) was adopted for statistical analysis. The data were analyzed using either one-way ANOVA when necessary or Student’s t-test. The results were expressed as mean ± SD. Statistically significant differences were identified at a *p*-value less than 0.05.

## Results

### Urolithin A Promotes Proliferation in HUVECs

**FIGURE 1:**
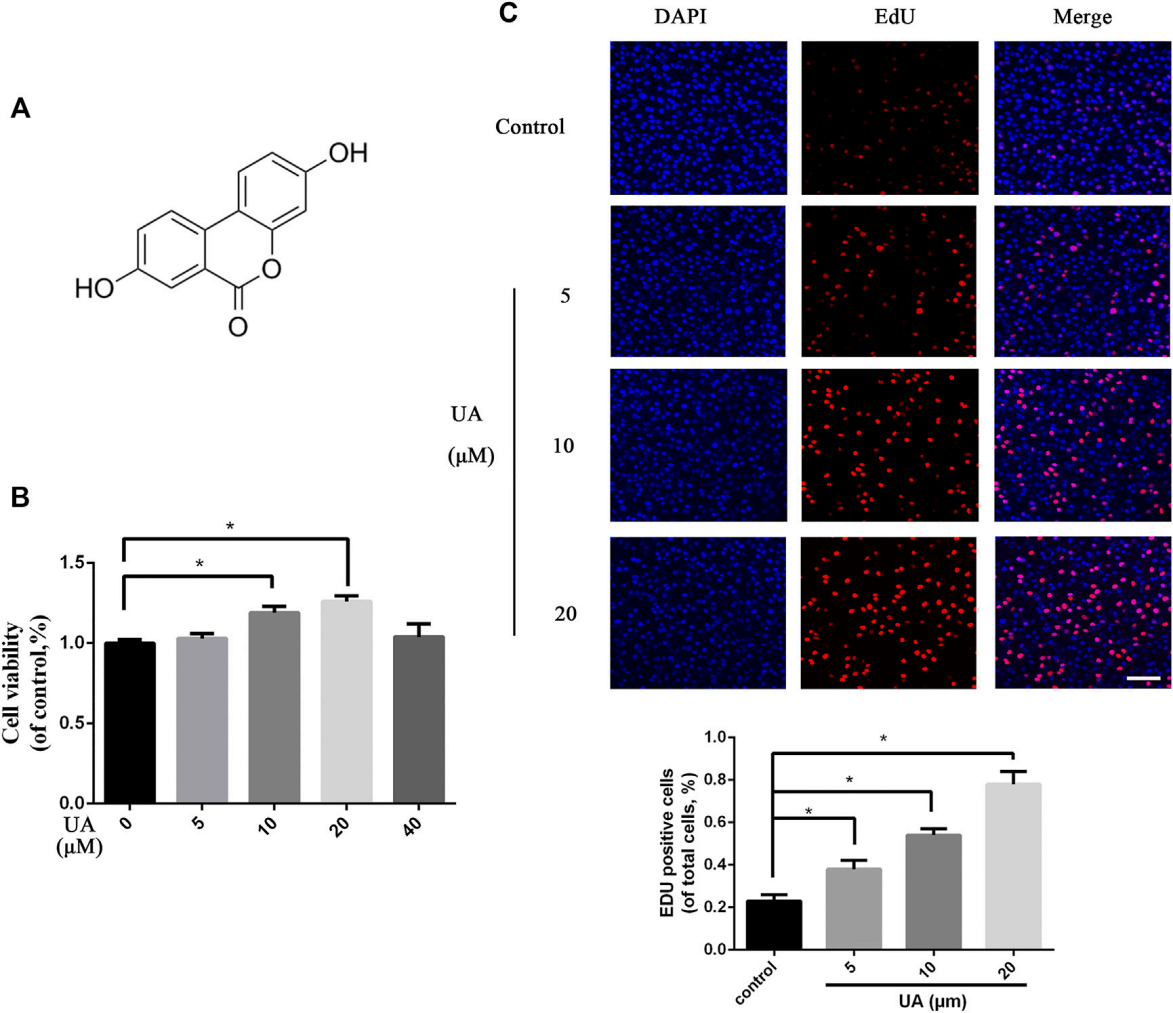
Effect of UA on cellular proliferation of HUVECs. **(A)** Chemical structure of UA. **(B)** Cell viability test results of HUVECs treated with different concentrations of UA. **(C)** EdU cell proliferation test results of HUVECs treated with different doses of UA (scale bar = 100 μm). Statistics of the EDU experiment. The data represent mean ± SD (*n* = 3) (*p < 0.05).

Figure 1A shows the molecular structure of UA. The viability test of UA on HUVECs was carried out using the CCK-8 test. HUVECs were seeded in a 96-well culture plate treated with different concentrations of UA (0, 5, 10, 20, 40 μM) for 48 h and then proceeded to the CCK-8 analysis. The results demonstrated increases in cell viability at 0–20 μM but not 40 μM of UA compared with the control group (Figure 1B). To better evaluate the protective effect of UA, we used the serum deprivation method and got the similar results to those in Figure 1B (Supplementary Figure S1). The EdU assay was used to investigate the cell proliferation capacity influenced by UA. The results confirmed that UA can promote the proliferation of HUVECs in a dose-dependent manner (Figure 1C).

### Urolithin A Enhances the Migration and Tube Formation Abilities in HUVECs

**FIGURE 2:**
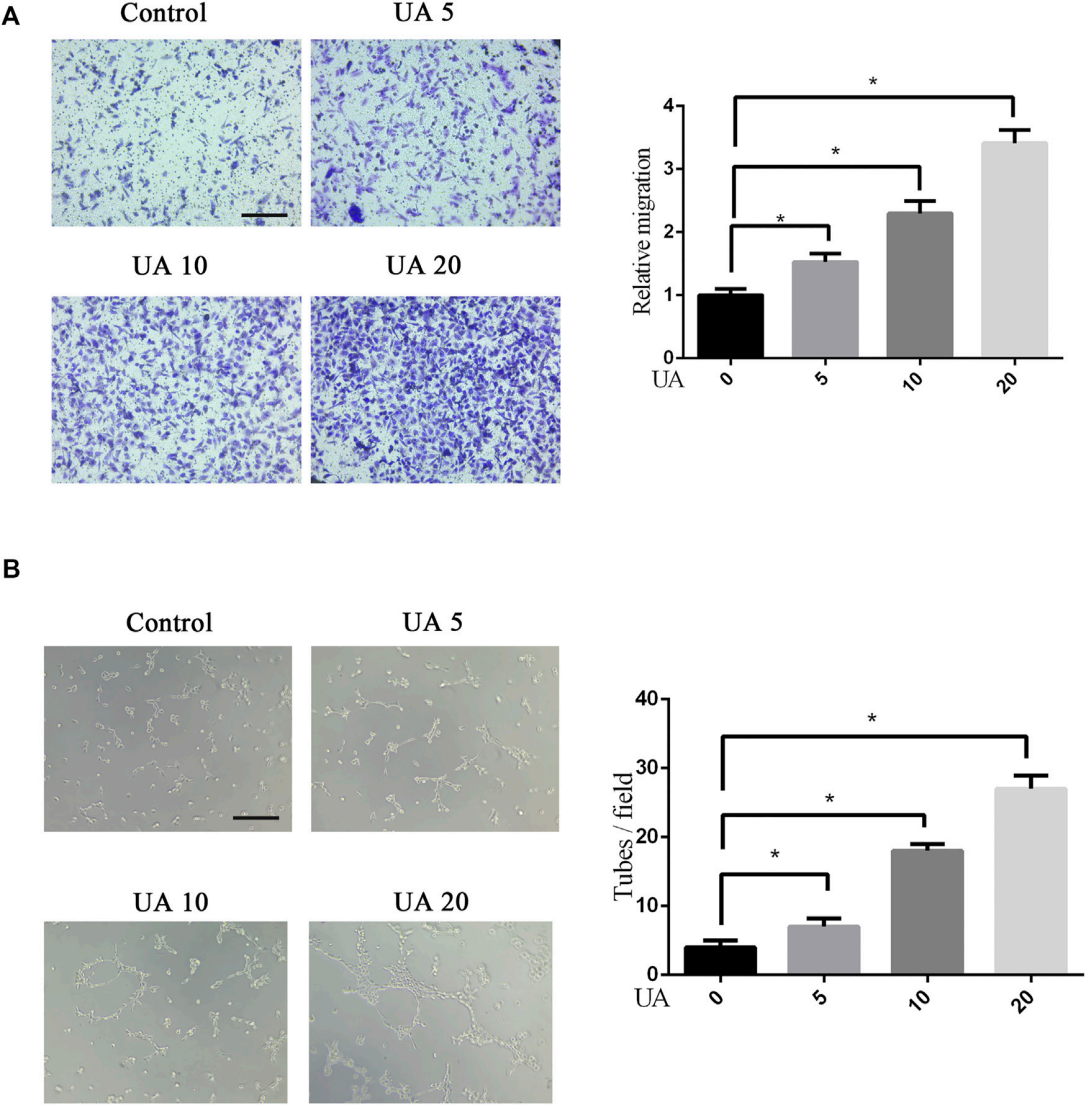
Effects of UA on cellular migration and tube formation of HUVECs. **(A)** Transwell chemotaxis test results of different treatments of HUVECs. The migration of HUVECs was enhanced after UA treatment (5, 10, and 20 μM UA). Statistics of the migration experiment. **(B)** After UA treatment, the tube-forming capacity of HUVECs is improved. Statistics of the tube-forming experiment. Scale bar = 100 μm. The data represent mean ± SD (*n* = 3) (*p < 0.05).

The targeted migration of endothelial cells plays a crucial role in the initial stage of angiogenesis (Wang et al., 2018b). Next, we used chemotaxis assays to detect the effect of UA on cell recruitment. The migration ability of HUVECs is enhanced in a dose-dependent manner (Figure 2A). In addition, *in vitro* angiogenesis experiments were used to study the tube-forming ability of HUVECs. As illustrated in Figure 2B, the UA treatment group, compared with the control group, significantly increased the number of HUVECs into tubes, indicating that UA can strongly promote the ability of HUVECs to form tubular structures.

### Urolithin A Inhibits PI3K/AKT Pathway in HUVECs

**FIGURE 3:**
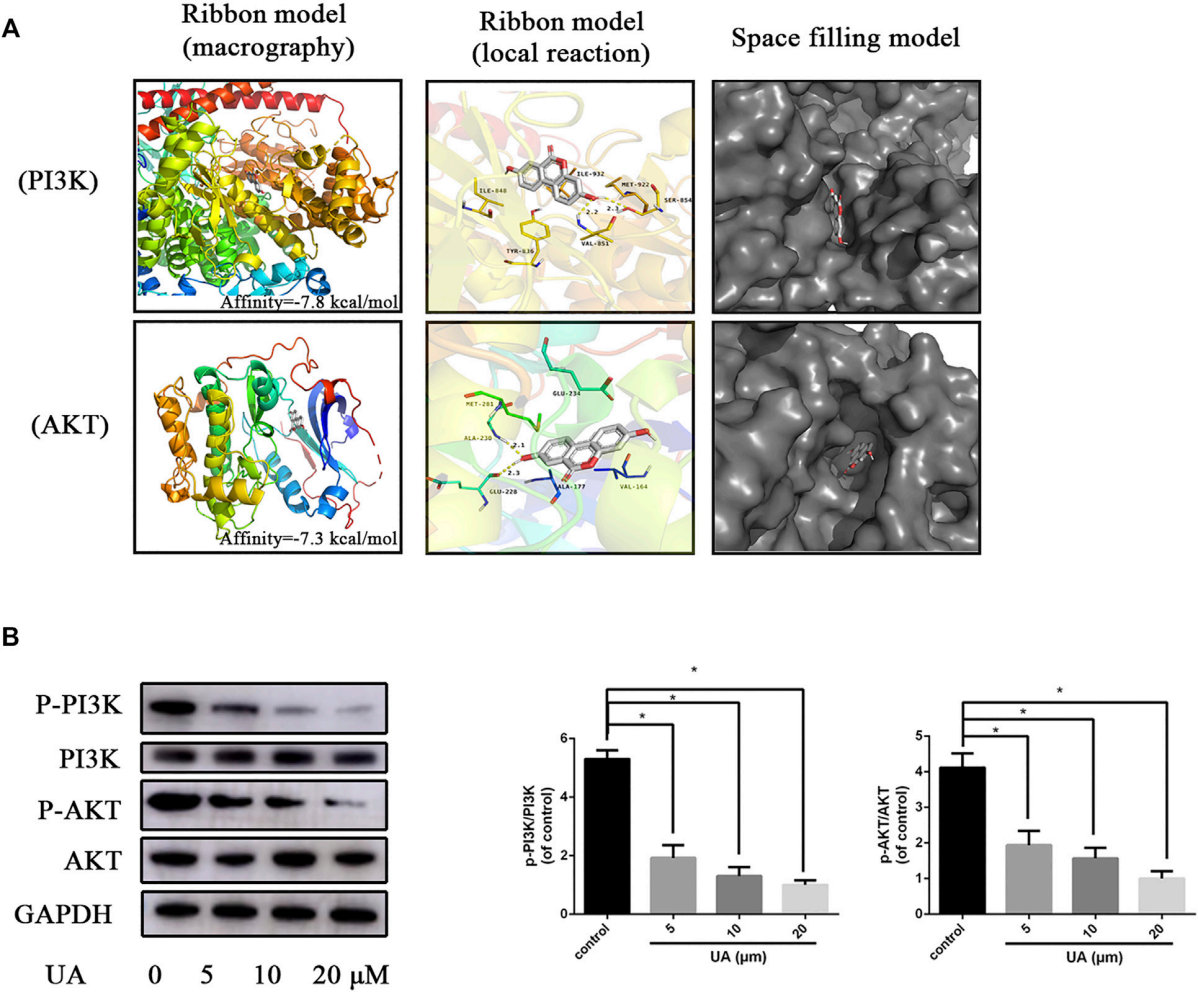
UA suppresses the PI3K/AKT pathway. **(A)** The protein residues are shown in the ribbon model and space-filling model. The local reaction of UA shows VAL-851 and SER-853 interactions with the PI3K structure. UA shows ALA-230 and GLU-228 interaction with the AKT structure. The space-filling model shows the binding of UA in the inhibitory binding pocket. **(B)** WB was used to detect the expression of PI3K and AKT proteins in HUVECs 48 h after UA treatment. Statistics of the WB experiment. The data represent mean ± SD (*n* = 3) (*p < 0.05).

To investigate whether UA has a direct affinity with PI3K and AKT, we performed molecular docking analysis of the molecular structure of UA with the protein structure of PI3K and AKT, according to respective binding pockets of the antagonist. Through the docking model, we discovered that UA formed some good connections and docked well in the binding sites of PI3K and AKT (Figure 3A). Moreover, the space-filling model directly showed the coverage of UA in the structure of respective proteins. Some important hydrogen bonds are formed between UA and PI3K including VAL-851 and SER-853, with a high affinity of −7.8 kcal/mol. Meanwhile, some hydrogen bonds are formed between UA and AKT including ALA-230 and GLU-228 with an affinity of -7.3 kcal/mol. To confirm the relationship between UA and related proteins, we performed WB experiments. *In vitro* experiments showed that the expression of P-PI3K and P-AKT proteins decreased in a concentration-dependent manner after UA treatment (Figure 3B). Collectively, UA may promote angiogenesis by regulating the PI3K/AKT pathway *in vitro*.

### Urolithin A Promotes Wound Healing in Rats

**FIGURE 4:**
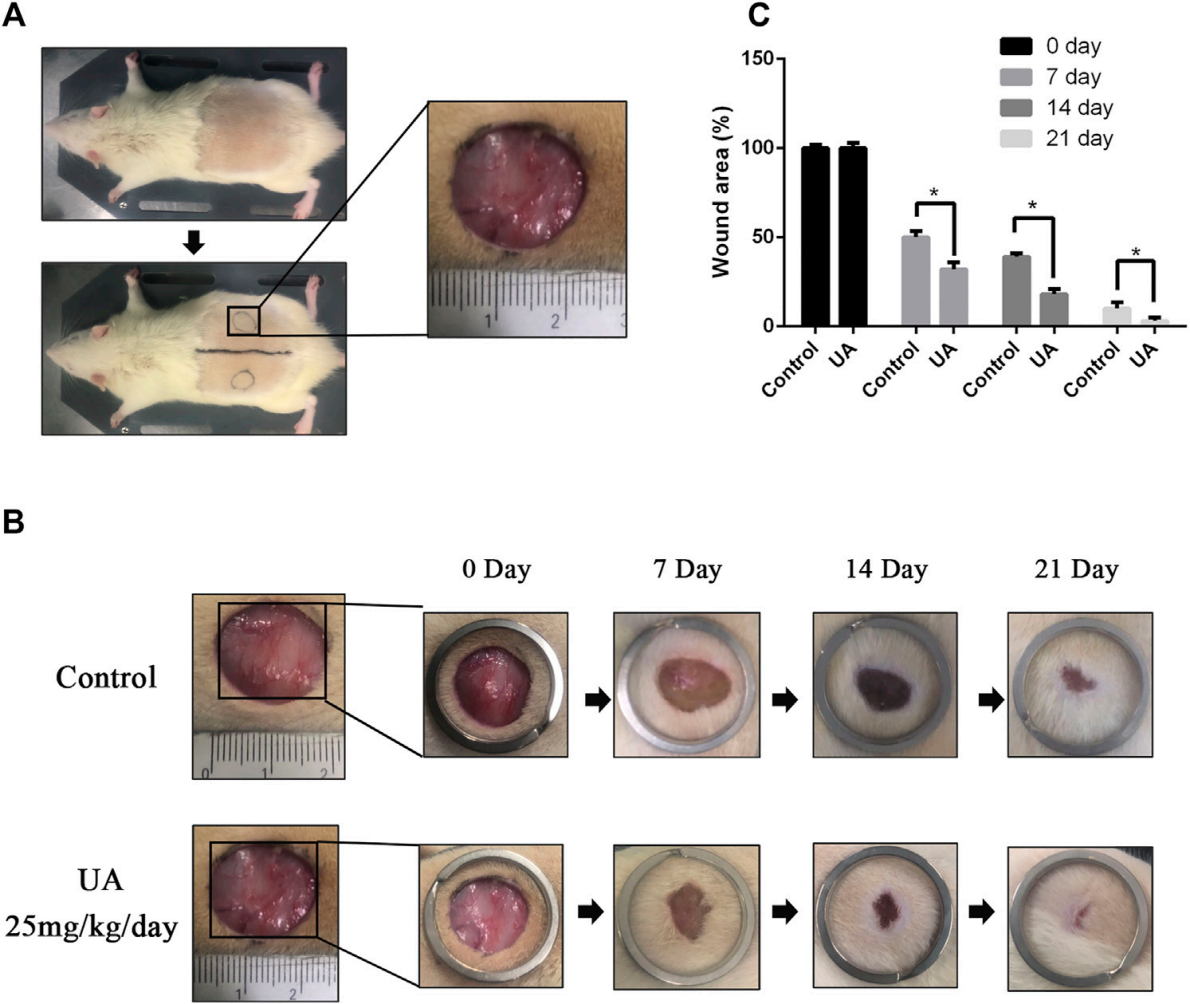
UA promotes wound healing *in vivo*. **(A)** A rough photo of the full-thickness wound model. **(B)** Representative images of the wound-healing process of rats treated with UA at different time points. **(C)** Wound-healing rates at different times. The data represent mean ± SD (*n* = 5) (*p < 0.05).

UA has the effect of promoting angiogenesis *in vitro*, and we supposed that UA may promote skin wound healing. Therefore, a full-thickness skin wound model was used to investigate whether UA has the ability of promoting wound repair. As shown in Figures 4A,B, wounds treated with UA healed more quickly than the control group. By day 7, the wound-healing rate of the UA treatment group was close to 70%, while the wound-healing rate of the control group was 50% (Figure 4C). On the 14th day, the healing speed of the UA group slowed down but was still significantly higher than that of the control group. On the 21st day, the wounds in the UA group were almost completely closed, while some wounds in the control group were still unhealed, indicating that UA accelerated wound healing.

### Urolithin A Promotes Collagen Deposition, Remodeling, and Cytokeratin Expression

**FIGURE 5:**
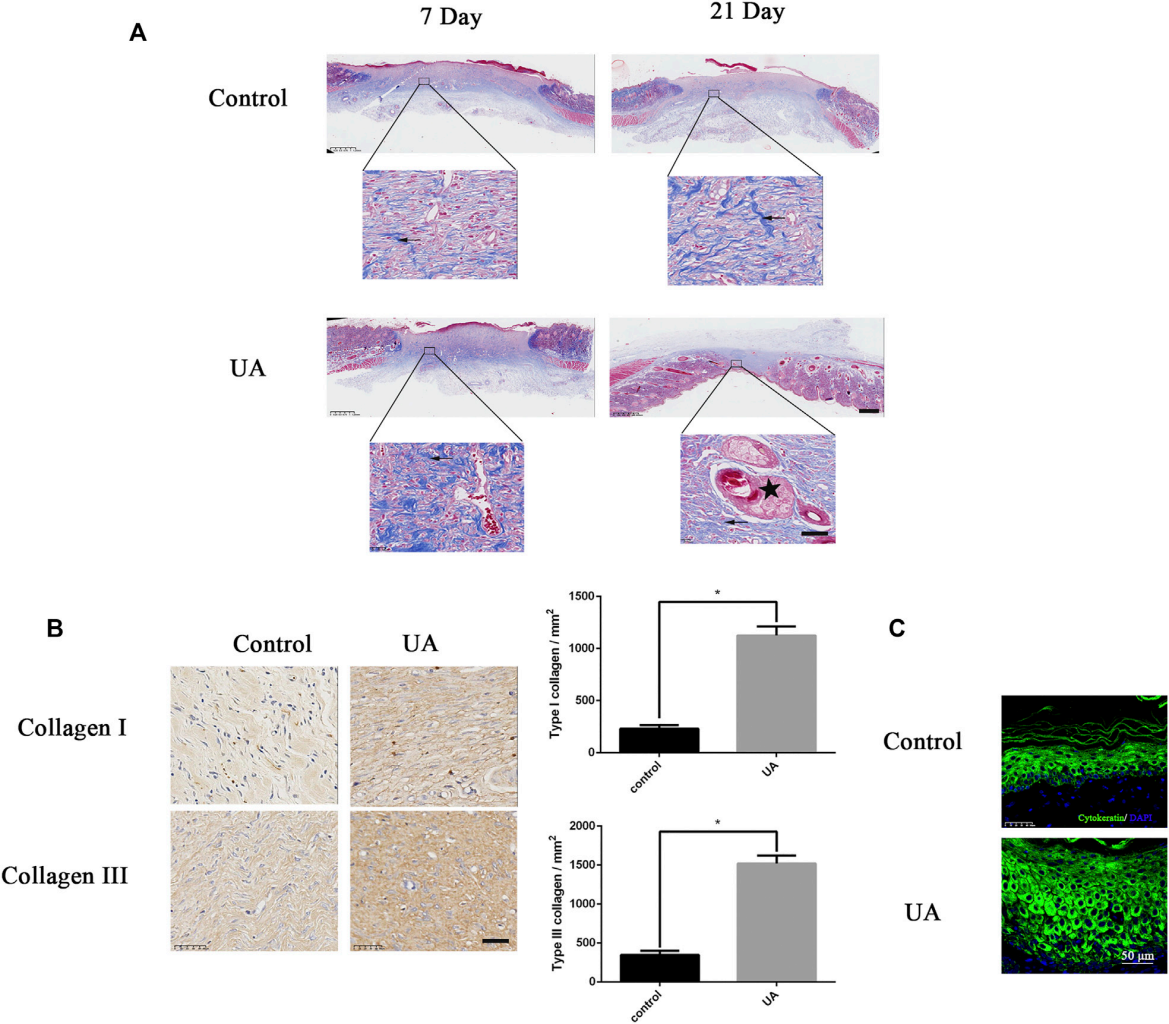
UA increases collagen synthesis and cytokeratin. **(A)** Masson trichrome staining of wound tissue on the 7th and 21st days after UA treatment (scale bar = 100 μm, 50 μm). Arrows represent collagen deposition, and the five-pointed star represents keratinization. **(B)** Representative images of type I and type III collagen immunohistochemical staining on the seventh day after surgery (scale bar = 50 μm). Statistics of immunohistochemistry experiments. **(C)** Representative fluorescence image of cytokeratin at the edge of the wound (scale bar = 50 μm). The data represent mean ± SD (*n* = 3) (*p < 0.05).

Next, we used Masson’s trichrome staining and type I and type III collagen immunohistochemical staining to evaluate collagen deposition and remodeling. Strong blue staining could be illustrated in the UA treatment group, showing regularly arranged regenerated collagen deposits (Figure 5A). Moreover, UA-treated wounds exhibited the significantly higher intensity of collagen I and III deposition than control (Figure 5B). We performed keratin immunofluorescence staining to assess the re-epithelialization status of the wound area. The neonatal epidermis is thicker in the UA treatment group than in the control group (Figure 5C), suggesting that the re-epithelialization rate of the UA treatment group was faster than that of the untreated group. Taken together, UA could accelerate collagen deposition, remodeling, and cytokeratin expression.

### Urolithin A Enhances Angiogenesis *In Vivo*

**FIGURE 6:**
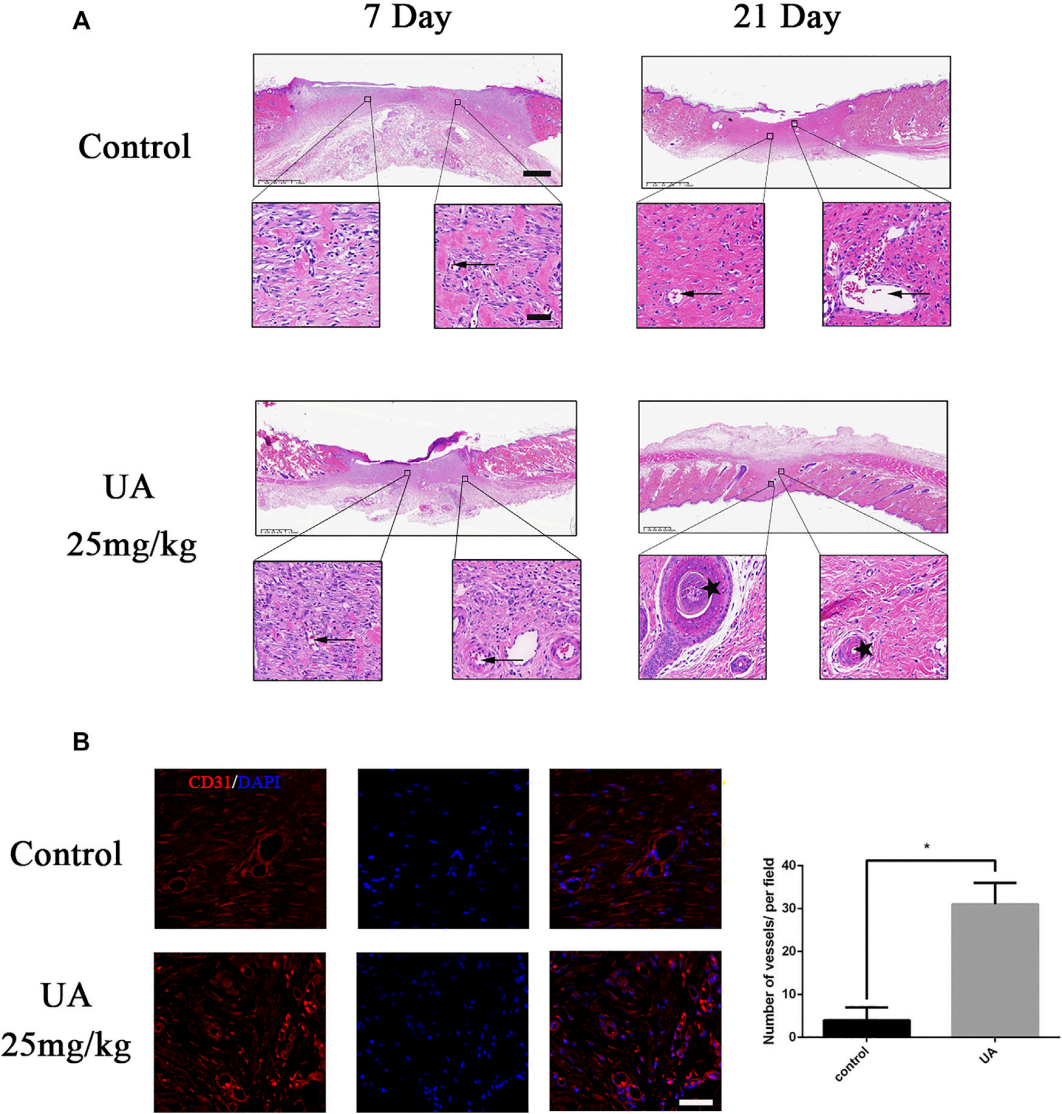
UA promotes angiogenesis *in vivo*. **(A)** HE staining of the wound surface at 7 and 21 days after operation (scale bar = 100 μm, 50 μm). Arrows represent blood vessels, and the five-pointed star represents keratinization. **(B)** Immunofluorescence staining images of CD31 (scale bar = 50 μm). Statistics of the immunofluorescence experiment. The data represent mean ± SD (*n* = 3) (*p < 0.05).

As shown in Figure 6A, after H&E staining, the number of blood vessels on the wound in the UA treatment group was significantly higher than that in the control group on day 7, indicating that UA treatment can significantly promote the formation of capillaries on the wound *in vivo*. On day 21, hair follicles have grown in the UA group. To evaluate wound angiogenesis, CD31 was used to characterize wound blood vessels. The number of new blood vessels on the seventh day in the UA group was significantly higher than that in the control group (Figure 6B). Collectively, UA could enhance the angiogenesis ability in wound-healing rats.

## Discussion

Rapid regenerative wound healing has always been a clinical challenge due to the high demands and complexity of the healing process, especially in chronic wounds (Gurtner, Werner, Barrandon, & Longaker, 2008). In the present study, we report that UA has a therapeutic effect on the repair of rat skin wounds by accelerating angiogenesis. Moreover, we found that UA enhanced HUVEC proliferation, migration, and tube formation abilities. The mechanism of UA promoting wound healing may be related to the inhibition of the PI3K/AKT signaling pathway. Our findings suggest that UA improves the biological functions of HUVECs and has a therapeutic effect on wounds.

To ensure adequate nutrition and oxygen supplementation for large-area wound defects and prevent cell death in ischemic areas, rapid and continuous vascularization is required, which is essential for effective skin wound regeneration (Martin, 1997). The vascular network is an important part of the dermis. Blood vessels are responsible for transporting oxygen and nutrients to skin cells (Amirsadeghi et al., 2020). Many tissue engineering materials, stem cells, and growth factors are applied to promote wound angiogenesis (Kumar & Kamalasanan, 2021; Lou et al., 2021). In general, the angiogenesis process can be divided into several stages, including endothelial cell proliferation, separation, migration, and differentiation (Lamalice and Le Boeuf, 2007). In our study, UA can significantly promote the proliferation of HUVECs, which can be considered a signal for the beginning of angiogenesis. In addition, UA-treated HUVECs significantly enhance their migration ability, which can benefit and accelerate the angiogenesis process, which is essential for the reconstruction of the vascular network. Ultimately, HUVECs differentiate into new capillaries, which is confirmed by the faster tube formation rate and the number of tubes. Taken together, UA shows a potent pro-angiogenesis effect *in vitro*.

It has been demonstrated that the PI3K/AKT pathway is an important cellular signaling pathway involved in a variety of cellular activities, including cell migration, proliferation, and survival (W. Dong et al., 2018; Guo et al., 2021; Zeng, He, Li, & Wang, 2021). Previous studies have indicated that PI3K could aggravate impairment of HUVECs and angiogenesis, indicating a potential target for wound healing (Hunter et al., 2019; M.; Zhang et al., 2020). AKT is a downstream signaling molecule of PI3K, which activates the PI3K/AKT pathway and can inhibit angiogenesis in cancer cells (R. Zhang et al., 2018). Regulating the PI3K pathway may be a new therapeutic target for accelerating skin regeneration and closing severely life-threatening wounds (Castilho et al., 2013). Akt/mTOR pathway dysfunction can lead to impaired wound healing in diabetic rats (Huang et al., 2015). In the present study, the molecular docking model showed that UA had a high affinity to PI3K and AKT which was proved by WB results. Taken together, our results illustrated that UA could facilitate angiogenesis via the PI3K/AKT signaling pathway.

To further elucidate the role of UA in wound repair, we used a full-thickness skin wound model. Immunohistochemistry and Masson staining showed that, in the process of wound healing, UA promoted collagen deposition and remodeling, which was characterized by a large number of collagen fibers arranged under the wound (Leivonen et al., 2005; Werner et al., 2007; Brem et al., 2009). During the healing process, the proper deposition of these two types of collagen fibers (collagen I and III) will facilitate wound healing and obtain a better healing area (Tang et al., 2020). In addition, the expression of cytokeratin, that is, the degree of keratinization, could increase its expression after UA treatment, indicating that UA could promote re-epithelialization of wound regeneration. HE and CD31 staining confirmed that UA increased the formation of new blood vessels in the wound-healing process. Angiogenesis partly depends on the synthesis of collagen, and cell proliferation is critical to the wound-healing process (Martin, 1997). The results of this study demonstrate that UA can promote angiogenesis in a full-thickness wound model. The rate at which UA regulates angiogenesis may help to accelerate collagen deposition and remodeling, ultimately leading to faster and better wound healing.

In summary, our research provides evidence that UA promotes angiogenesis by inhibiting the PI3K/AKT signaling pathway. In addition, UA increases blood vessel formation, collagen deposition, and remodeling, all of which accelerate skin wound healing. These results indicate that UA has great therapeutic potential for endothelial injury–mediated vascular diseases, especially for patients with skin wounds.

## Data Availability

The original contributions presented in the study are included in the article/Supplementary Material, further inquiries can be directed to the corresponding authors.
