# Supplementary material for: Urolithin A Promotes Angiogenesis and Tissue Regeneration in a Full-Thickness Cutaneous Wound Model
Source: Front Pharmacol. 2022 Mar 14;13:806284. doi: 10.3389/fphar.2022.806284 (PMC8964070; doi:10.3389/fphar.2022.806284)
Supplement: Supplementary file 1 [file Image1.pdf]

Supplemental Information

Urolithin A promotes angiogenesis and tissue regeneration in a full-thickness cutaneous wound model

Running title：Urolithin A in wound healing

Zhen-hua Feng[1,2 *], Jia Chen[1,2*], Pu-tao Yuan[1,2 *], Zhong-yin Ji[1,2], Si-yue Tao[1,2], Lin Zheng[1,2], Xiao-an Wei[1,2], Ze-yu Zheng[1,2], Bing-jie Zheng[1,2], Bin Chen[3#], Jian Chen[1,2#], Feng-dong Zhao[1,2#]

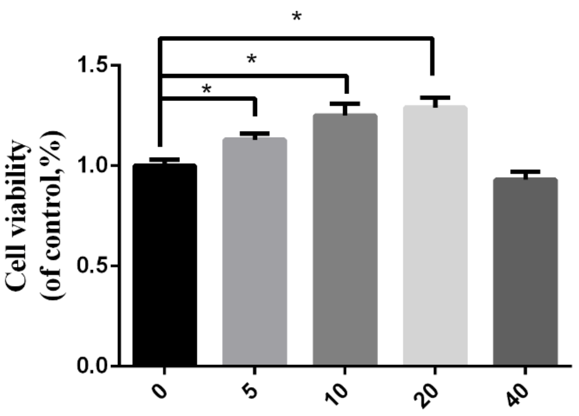

Supplementary Fig.1 Cell viability test results of HUVECs treated with different concentrations of UA in serum-free medium.
